# Improved Strength and Toughness of Carbon Woven Fabric Composites with Functionalized MWCNTs

**DOI:** 10.3390/ma7064640

**Published:** 2014-06-18

**Authors:** Eslam Soliman, Usama Kandil, Mahmoud Reda Taha

**Affiliations:** 1 Department of Civil Engineering, Assiut University, Assiut 71515, Egypt; E-Mail: eslam.soliman@eng.au.edu.eg; 2 Polymer Nanocomposites Center, Egyptian Petroleum Research Institute, 1 Ahmed El-Zomor Street, Nasr City, Cairo 11727, Egypt; E-Mail: alfa_olefins@yahoo.com; 3 Department of Civil Engineering, University of New Mexico, MSC01 1070, Albuquerque, NM 87131, USA

**Keywords:** carbon nanotubes, functionalization, woven fabric, epoxy, FTIR, toughness

## Abstract

This investigation examines the role of carboxyl functionalized multi-walled carbon nanotubes (COOH-MWCNTs) in the on- and off-axis flexure and the shear responses of thin carbon woven fabric composite plates. The chemically functionalized COOH-MWCNTs were used to fabricate epoxy nanocomposites and, subsequently, carbon woven fabric plates to be tested on flexure and shear. In addition to the neat epoxy, three loadings of COOH-MWCNTs were examined: 0.5 wt%, 1.0 wt% and 1.5 wt% of epoxy. While no significant statistical difference in the flexure response of the on-axis specimens was observed, significant increases in the flexure strength, modulus and toughness of the off-axis specimens were observed. The average increase in flexure strength and flexure modulus with the addition of 1.5 wt% COOH-MWCNTs improved by 28% and 19%, respectively. Finite element modeling is used to demonstrate fiber domination in on-axis flexure behavior and matrix domination in off-axis flexure behavior. Furthermore, the 1.5 wt% COOH-MWCNTs increased the toughness of carbon woven composites tested on shear by 33%. Microstructural investigation using Fourier Transform Infrared Spectroscopy (FTIR) proves the existence of chemical bonds between the COOH-MWCNTs and the epoxy matrix.

## 1. Introduction

The use of fiber-reinforced polymer (FRP) composites has grown very rapidly over the last few decades, due to their attractive physical, mechanical and thermal properties [1]. Some of their common applications include civil infrastructure, composite bridge decks, oil and gas pipelines and turbine blades in windmills. The woven fabric composite, in particular, has attracted the interest of engineers and researchers, due to its relatively high delamination and impact resistance. However, the failure of FRPs is complex, especially when multi-layer laminates are examined. The failure behavior depends on many factors, such as the loading nature and direction with respect to the fibers, the fabrication quality and the structure of the composite fabric. Typical failure modes of FRPs include fiber fracture, matrix fracture, fiber-matrix interface debonding and interlaminar delamination [2,3]. One of the recent promising techniques to overcome the premature failure of composites due to delamination/debonding is to reinforce the composites with nanoparticles.

Carbon nanotubes (CNTs), nano-clay, graphene nanoparticles (GNPs), and nano-silica are common non-metallic nanoparticles used to fabricate nanocomposites [4,5]. Discovered by Ijima in 1991 [6], CNTs have attracted many scientists worldwide, because of their distinguished mechanical properties compared with conventional structural materials. Therefore, many researchers have focused on using the CNTs in polymer nanocomposites. Over the last two decades, various techniques have been developed to ensure a good dispersion of CNTs in polymeric matrices, such as non-covalent [7,8,9] and covalent [10,11,12] functionalization. The covalent functionalization involves impregnating functional groups on the surface of nanotubes. The functional groups are expected to react with the polymer matrix and increase the interfacial bond significantly. A review for the use of functionalized CNTs in polymeric matrices can be found elsewhere [13,14,15].

Several studies reported good improvement in FRP mechanical properties when CNTs were used in polymer nanocomposites. The mechanism of CNTs in composite is expected to resist the inter-fiber fracture and enhance their respective mechanical properties. The improvements of FRP composites include the tensile and shear property of glass fiber-reinforced polymer (GFRP) composites [16] and Mode I and Mode II fracture toughness [17,18]. For instance, Qiu *et al.* [16] examined the tensile and shear behavior of (GFRP) composites. With 1.0% by weight functionalized multi-walled carbon nanotubes (MWCNTs), they reported a 14% and 5% increase in on-axis tensile and shear strengths, respectively. They also reported a 20% and 8% increase in tensile Young’s and short beam shear moduli. In addition, Garcia *et al.* [18] examined the fracture toughness of carbon fiber-reinforced polymer (CFRP) with nanotubes joining the prepregs, and they found a 250% increase in Mode I and a 300% increase in Mode II fracture toughness. Furthermore, compression shear tests were performed to determine the interlaminar shear strength (ILSS) of glass woven fabric composites with different dispersion techniques and showed no variation in ILSS due to the addition of 0.5 wt% MWCNTs [19].

Few studies examined using nanoparticles to improve the flexure behavior of FRP composites, such as increasing the ultimate strength, the flexural modulus or the energy absorption under flexural loads. Hossain *et al.* [20] reported a 49% and 31% increase in the flexural strength and modulus of on-axis woven E-glass/polyester composites reinforced by 0.1–0.4 wt% carbon nanofibers (CNF). In addition, 1.5 wt% aligned MWCNTs are used to improve on-axis flexure strength and modulus for (CFRP) composites by 74% and 75%, respectively [21]. To date, the effect of using functionalized MWCNTs on the flexure behavior of carbon woven fabric composites has not yet been reported. In this investigation, a carboxyl functionalized multi-walled carbon nanotube (COOH-MWCNTs) epoxy nanocomposite is used to improve the on- and off-axis flexural behavior of woven carbon fiber composites.

## 2. Experimental Methods

### 2.1. Materials

The carbon fiber and the epoxy were supplied by U.S. Composites, Inc., Florida, FL, USA. The fabric is FG-CARB5750, a balanced plain bidirectional (0°–90°) weave, PAN-based fiber, a 3 k tow size and 254 µm-thick. The tensile strength of the raw carbon fibers is 4.48 GPa and the tensile modulus is 231 GPa. The bi-directional plain weave fabric was selected over the other types of weaves (*i.e.*, twill or satin), because it provides the largest number of interlaces between the warp and filling threads. Such a large number of interlaces maintains the geometry of the woven fabric cloth and minimizes any weaving of the fiber bundles that might occur due to rolling the epoxy during the fabrication process of the FRP composites.

The epoxy used in fabrication is the commercially available EPOTUF^®^ 37-127 epoxy system. The epoxy resin is a diluted liquid based on bisphenol-A and contains diglycidyl ether (DGEBPA), while the hardener is an aliphatic amine. The resin to hardener mixing ratio was 2:1; the pot life was 30–45 min at 80 °F (26.7 °C); the set time was 5–6 h, and the curing time was 24–28 h at room temperature. This type of epoxy has relatively low viscosity, which facilitates the impregnation of the carbon fibers during the fabrication process. Furthermore, the epoxy pot life is relatively long to allow adequate time for the impregnation and the application of the vacuum environment before the epoxy hardens partially. The tensile strength and tensile elongation for the epoxy are 65 MPa and 25%, respectively.

Carboxyl functionalized COOH-MWCNTs were supplied by Cheap Tubes, Inc., Vermont, VT, USA. According to the manufacturer’s data, the outer diameter of the CNTs is 20–30 nm, the inner diameter is 5–10 nm and the length is 10–30 µm with an aspect ratio ranging from 500 to 1000. The nanotubes were manufactured using the catalytic chemical vapor deposition technique with the purity being greater than 95 wt%. The functionalization was performed by the manufacturers using a mixture of non-organic acids. According to the manufacturer, the functionalized MWCNTs had (COOH) functional groups of 1.23 wt%.

### 2.2. COOH-MWCNTs/Epoxy Nanocomposite Fabrication

**Figure 1:**
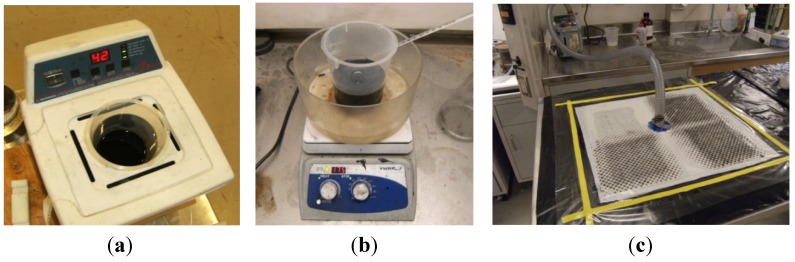
Fabrication of multi-scale epoxy/carbon woven fabric composite specimens: (**a**) sonication of COOH-MWCNTs in the epoxy resin; (**b**) stirring of the COOH-MWCNTs in the epoxy resin; and (**c**) the vacuum environment applied to the composite specimens during the curing of the epoxy.

**Figure 2:**
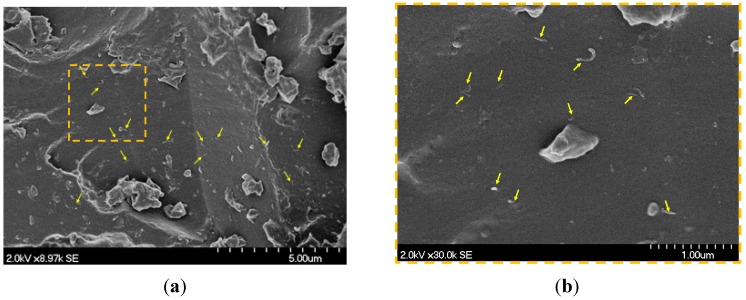
SEM image showing the dispersion of 1.0 wt% COOH-MWCNTs in the epoxy matrix. (**a**) The general view with the marked area shown at high resolution in (**b**); (**b**) the high resolution view of COOH-MWCNTs in epoxy showing individual carbon nanotubes.

Two different types of treated epoxies were produced to fabricate the carbon woven composites in this study. The first type was a neat epoxy prepared by mixing the resin with the hardener and applying the mixture directly to the carbon fabric during the impregnation process. The second type was prepared by reinforcing the epoxy with functionalized COOH-MWCNTs. The COOH-MWCNTs were added first to the resin, and the mixture was sonicated for 1.0 h at 40 °C, as shown in Figure 1a. In order to ensure the chemical reaction between the functional groups on the surface of the nanotubes and the resin chains, the dispersed mixture was stirred for 2.0 h at 80 °C. The hardener was added after cooling the resin, and the COOH-MWCNTs/epoxy nanocomposite was further homogenized by mechanical mixing for 10 min and then used in the composite fabrication. Figure 2a shows the Scanning Electron Microscope (SEM) images for well-dispersed 1.0 wt% COOH-MWCNTs in the epoxy matrix. The image shows the absence of aggregates/agglomeration of MWCNTs as an indication of the effectiveness of the dispersion process using sonication and mechanical stirring. A close view is shown in Figure 2b confirming the diameter and their good dispersion of the MWCNTs.

### 2.3. Fabrication of the Multi-Scale Woven Fabric Epoxy Composite

Fabrication of the woven fabric composite plates was performed in accordance with ASTM D5687 [22]. The vacuum-assisted hand lay-up technique was implemented to fabricate the composite plates. Ten layers of fabric were used to fabricate the specimens. The average dimensions of the composite specimens were 40 mm-long, 19 mm-wide and 2.7 mm-thick. Peel plies, non-porous and porous release films, breather plies and an aluminum plate were used to ensure straight and compacted composite sheets and to facilitate the peeling off of the composite after curing. Adequate epoxy impregnation was achieved using a roller, and a breather ply was placed to form the uniform air paths once the vacuum was applied. The same epoxy/carbon fabric weight ratio was used during the fabrication of all composite specimens in order to maintain the same epoxy weight fraction. The epoxy weight fractions were obtained by measuring the change in weight between the original carbon fabric before and after epoxy impregnation and ranged between 30% and 33%. It is important to note that the addition of COOH-MWCNTs increased the viscosity of the epoxy during the fabrication; therefore, the limitation on the upper bound of the COOH-MWCNTs content was set as 1.5 wt% of epoxy. The vacuum bag contents were maintained at a vacuum level of 2.3 × 10^−2^ Torr for 24 h. This relatively high level of vacuum applied for long time period ensures the elimination of all air bubbles. Furthermore, to ensure the consistent production of the composite with different levels of nanomaterials, ASTM D3171 was used for determining the fiber weight fraction, and the fiber volume fractions for the different composite materials were found to be 54.3%, 55.5%, 53% and 52.4% for the neat, 0.5%, 1.0% and 1.5% COOH-MWCNTs epoxy woven fabric composites, respectively, confirming the acceptable consistency in production [23]. Relatively high void contents of 2%, 2.3%, 1.5% and 3% were observed in 0, 0.5 wt%, 1.0 wt% and 1.5 wt% COOH-MWCNTs, respectively, due to the use of the vacuum-assisted hand layup technique in the fabrication process. The variation in void content is ±0.8%, and the variation in the fiber fraction is ±1.7%. Such variation in void and fiber fractions within ±2.0% was reported in the literature to have an insignificant effect on the mechanical properties of composites [24,25,26]. Therefore, the above variations would not affect our ability to examine the significance of COOH-MWCNTs on the strength and toughness of carbon fiber composites.

### 2.4. Mechanical Testing

**Figure 3:**
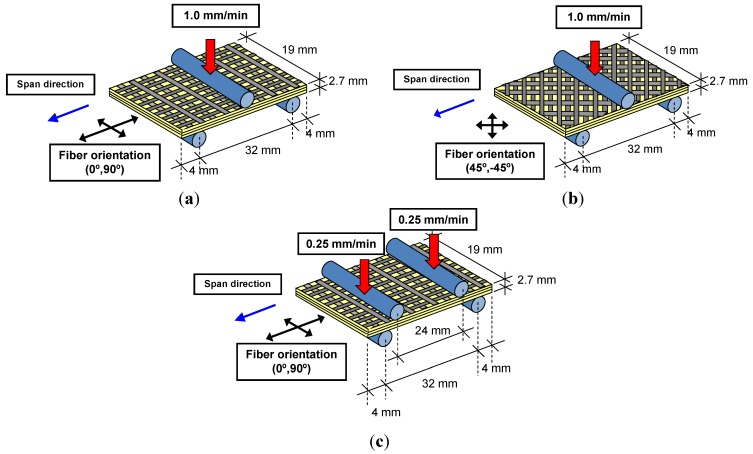
on- and off-axis arrangements for a flexure test: (**a**) on-axis flexure test; (**b**) off-axis flexure test; (**c**) on-axis shear test.

Three-point bending tests were conducted to examine the on- and off-axis flexure behavior of the composite specimens. The flexure test setup and geometry of the specimens are shown in Figure 3a,b. In the on-axis test, the orientation of the fibers was in the span direction, while the fibers were oriented at a 45° angle with respect to the span direction in the off-axis test. On the other hand, the short-span shear test was conducted on the on-axis composite plates via four-point bending. The shear test setup is shown in Figure 3c. The flexure and shear tests were performed on the composite plates using an MTS^®^ Bionix servo hydraulic system. The machine has a load cell with a capacity of 25 kN and a maximum stroke of 130 mm. The sampling rate for all of the experiments was 50 Hz, and the data was collected by the FlexStar MTS^®^ 793 data acquisition system. In each test, the load and displacement were measured and recorded.

The flexure stress-strains σ_*f*_ − ε_*f*_ were then obtained using Equations (1) and (2) following ASTM D790-10 [27].

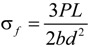
(1)

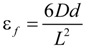
(2)
where *P* is the applied load, *L* is the span length, *b* is the width of the specimen, *d* is the depth of the specimen and *D* is the deflection of the mid-span. On the other hand, the average shear stress-strains τ − γ were obtained according to Equations (3) and (4).

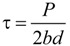
(3)

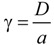
(4)
where *a* is the shear span. In addition to the neat epoxy, three loadings of COOH-MWCNTs were examined. They are 0.5, 1.0 and 1.5 wt% of epoxy. Three specimens were tested for each COOH-MWCNTs loading. The flexure and shear strength, modulus and toughness were then evaluated and compared. Statistical analyses were performed on the test results and the statistical significance of the difference between the specimens was evaluated using the Student’s *t*-test assuming a 95% level of significance.

### 2.5. Microstructural Investigation

To explain the role of functionalized COOH-MWCNTs on the behavior of carbon fibers, Fourier Transform Infrared Spectroscopy (FTIR) measurements were conducted on 25.4 mm × 25.4 mm samples cut from CFRP specimens. Three types of samples were compared: neat CFRP samples, CFRP samples incorporating 0.5 wt% COOH-MWCNTs and CFRP samples incorporating 1 wt% COOH-MWCNTs. Furthermore, COOH-MWCNTs powder was separately measured. All of the samples were analyzed with biconical reflectance Nicolet Nexus 670 micro-Fourier Transform Infrared Spectroscopy (Micro-FTIR) (Thermo Fisher Scientific, Waltham, MA, USA). The FTIR has a continuum microscope with a Globar source, an XT-KBr beam splitter and a MCT-A detector over a 100 × 100 micron area with a 4 cm-1 resolution. Spectra were background corrected using a reflective gold slide and converted to absorbance using the Kramers-Kronig equation, as per the standard FTIR analysis method after Stefanie *et al.* [28].

### 2.6. Micromechanical Modeling

In order to understand the failure mode of the on- and off-axis flexure tests, a micromechanical finite element model is developed under ANSYS^®^ APDL modeling environment (Pennsylvania, PA, USA) coupled with micromechanical Autodesk^®^ composite simulation analysis (California, CA, USA). The micromechanical model is based on multi-continuum theory (MCT) for composite analysis. In MCT, continuum-based (phase-averaged) stresses and strains can be predicted for each constituent (e.g., fibers or matrix) in finite element (FE) analysis. In addition, the failure criterion for each constituent of the composite is predicted and displayed separately, allowing a progressive failure to take place. Previous research proved the failure analysis by MCT to be fairly close to experimental observations, since it takes into account matrix damage and the associated reduction in stiffness [29,30,31]. Two 3D FE models for flexure test specimens were constructed. The two models consist of 10 layers of woven fabric composite with an equal fill and wrap fiber volume fraction and a thickness of 0.27 mm. In the first model, the fill fibers were set in the span direction, while in the second model, the fill fibers were set at a 45° angle with respect to the span direction. 3D SOLSH190 elements are used to model the laminated composite. Table 1 lists all material properties used in the FE simulations. The material properties of the constituent materials are obtained experimentally [32], from the manufacturer’s data or using classical lamination theory (CLT).

**Table 1:** The material properties of woven fabric composite laminates used in FE simulation.

Designation	*E*_11_, GPa	*E*_22_, GPa	*G*_12_, GPa	+*S*_11_, MPa	−*S*_11_, MPa	*S*_12_, MPa	*υ*_12_
Lamina	40 *	40 *	3.9 *	555 *	−310 †	70 *	0.20 †
Fibers	230 †	50 †	–	–	–	–	–
Matrix	2.0 *		0.74 ‡	–	–	–	0.35 †

* The material properties obtained experimentally from [32]; † the material properties obtained from the manufacturer’s specifications and other sources; ‡ the material properties computed using classical lamination theory (CLT); *E*_11_: elastic modulus in the fill fiber direction; *E*_22_: elastic modulus in the wrap fiber direction; *G*_12_: in-plane shear modulus; +*S*_11_: ultimate tensile strength in the fill or wrap fiber directions; −*S*_11_: ultimate compressive strength in the fill or wrap fiber directions; *S*_12_: in-plane shear strength.

## 3. Results and Discussion

**Figure 4:**
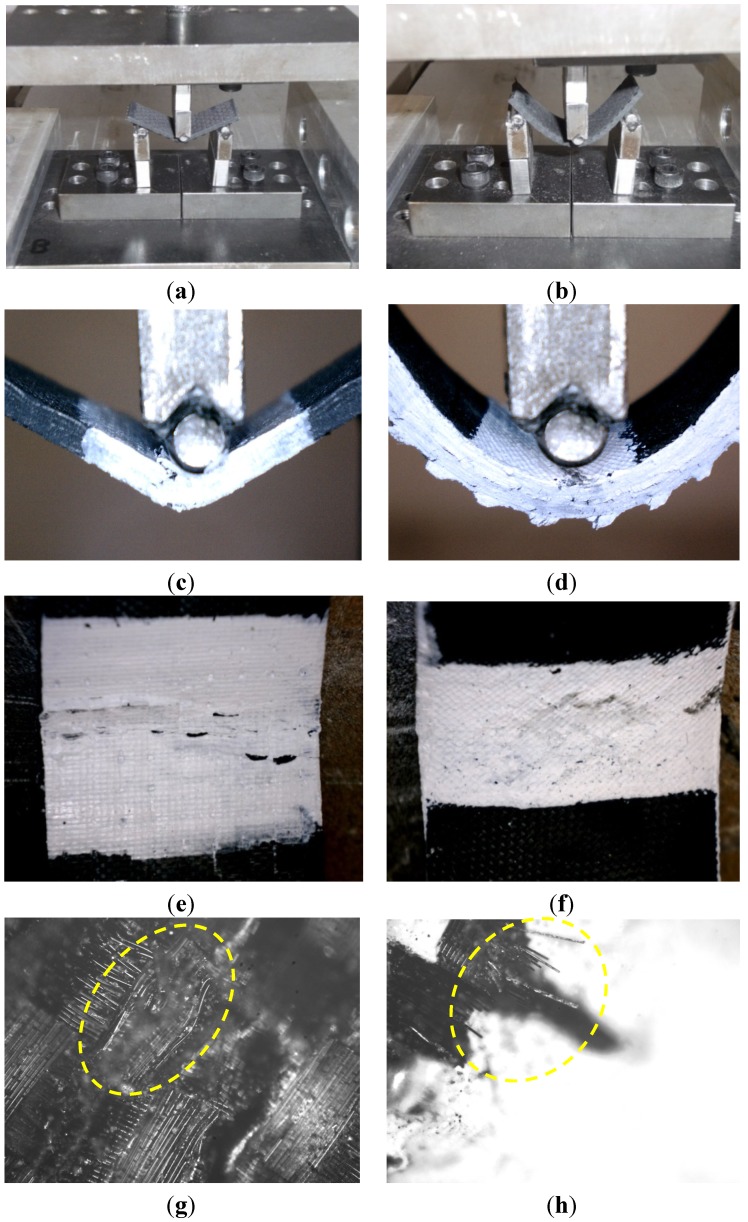
Deformation and failure of on- and off-axis flexure composite specimens. (**a**) deformation of on-axis flexure specimens; (**b**) deformation of off-axis flexure specimens; (**c**) failure of on-axis flexure specimens; (**d**) failure of off-axis flexure specimens; (**e**) kink/breakage of fibers at the top surface; (**f**) shear-off of the fibers at the top surface; (**g**) high resolution microscopic image for the kink of fibers at the top surface of the on-axis test; (**h**) high resolution microscopic image for the rupture of fibers at the bottom surface of the on-axis test.

Figure 4a,b shows the deformed shape of the on- and off-axis flexure tests, respectively. As expected, the off-axis specimens exhibited higher deflection and less strength than the on-axis specimens. Close views for the failed on-axis specimens show that failure occurred due to kink/breakage of the fibers in the compression side (Figure 4c,e). This is expected, since FRP composites are stronger in tension than they are in compression. In addition, high resolution microscopic images in Figure 4g,h show the kink and rupture of fibers in the top and bottom surfaces of the on-axis test specimens, respectively. On the other hand, failure of the off-axis specimens was due to shear-off of the fibers at a 45° angle in the compression zone, as shown in (Figure 4d,f). This shear-off is attributed to the weak shear strength of the epoxy matrix.

**Figure 5:**
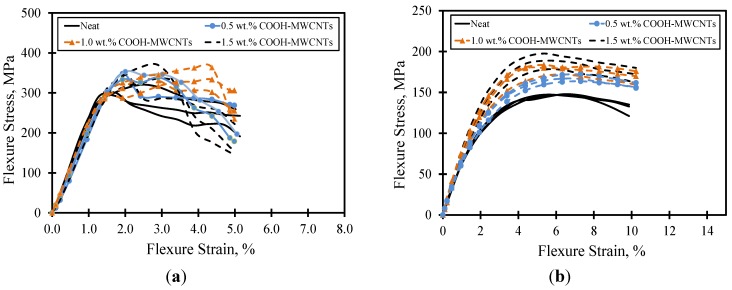
Flexure stress-strain curves for composite plates. (**a**) on-axis; (**b**) off-axis.

The flexure stress-strain curves for the on- and off-axis flexure tests are depicted in Figure 5a,b up to the 5% and 10% strain levels, respectively. For all COOH-MWCNTs loadings, the on-axis flexure stress-strain curves were linear up to a maximum value corresponding to the flexure strength of the composite specimens, where the failure in the compression side occurred (Figure 5a). Once compression failure occurred, the flexure stress was reduced as the fiber breakage propagated through the thickness. The linearity in the on-axis stress strain curve before failure proves the fiber domination of the on-axis flexure behavior. On the contrary, a non-linear stress-strain curve associated with a yield-like plateau was observed in the off-axis flexural behavior (Figure 5b). The significant non-linearity in the off-axis stress-strain curves is attributed to the matrix domination on the off-axis flexural behavior. In addition, the post-peak loss in flexure stress occurred in a more gradual fashion in the off-axis behavior than it occurred in the on-axis direction.

Table 2 and Table 3 show the statistical analyses for the mechanical properties of on- and off-axis composite specimens with various COOH-MWCNTs loadings. The flexure toughness for the on-axis test specimens were computed from the area under the stress-strain curves up to the 3.0% flexure strain level; the limit sufficiently extends beyond the fiber breakage first observed at the end of the proportional limit. On the other hand, the flexure toughness for the off-axis specimens were computed from the area under the stress-strain curves up to the 5.0% strain level, The proposed limits meet the recommendations of ASTM D790 [27]. From both tables, it can be observed that the flexure strength (~150–180 MPa) and modulus (~6–8 GPa) of the off-axis flexure test specimens were about one-half and one-third of that of the on-axis flexure test specimens, respectively. This is a typical observation supported by composite theory, where the behavior in the on-axis direction dominated by the strong fibers is much stronger than off-axis direction dominated by the matrix. The coefficients of variation for all specimens were below 10%, and therefore, all cases of COOH-MWCNTs loadings showed valid results.

Based on the Student’s *t-*test, no significant statistical difference was observed in the on-axis flexure strength, modulus and toughness between composite specimens with various COOH-MWCNTs loadings and the neat material. This observation confirms that the on-axis flexural behavior of the composite plates is dominated by the strong carbon fibers oriented in the span direction, and the change/modification in the epoxy matrix produces minimal to no effect on the on-axis behavior. A similar observation related to the fiber domination on the behavior of woven fabric composites subjected to on-axis tension was reported earlier by Naik *et al.* [33] and recently by Soliman *et al.* [32]. In this case, the composite coupons exhibit fiber breakage associated with limited damage in the matrix. On the other hand, significant statistical differences in the flexure strength, modulus and toughness associated with the off-axis flexural test were observed especially with the addition of large contents of COOH-MWCNTs. For instance, improvements in flexure strength, modulus and toughness with the addition of 1.5 wt% COOH-MWCNTs reached 19%, 28% and 20.7%, respectively (Table 3).

**Table 2:** Statistical analyses for the on-axis flexure test with various COOH-MWCNTs loadings: flexure modulus of elasticity (*E*), flexure strength (σ_u_) and flexure toughness at the 3% strain level (*T*-3%).

COOH-MWCNTs	Criterion	*E* (GPa)	σ_u_ (MPa)	*T*-3% (MPa.mm/mm)
0 wt% (neat)	Mean	21.70	320	6.70
	STD	0.7	11.93	0.25
0.5 wt%	Mean (% increase)	20.40 (−6%)	348 (8%)	6.96 (3.9%)
	STD	0.61	6.24	0.52
1.0 wt%	Mean (% increase)	19.33 (−10%)	340 (6%)	6.77 (1.04%)
	STD	0.85	23.90	0.45
1.5 wt%	Mean (% increase)	20.40 (−6%)	344 (7%)	6.94 (3.48%)
	STD	0.82	29.28	0.36

**Table 3:** Statistical analyses for the off-axis flexure test with various MWCNTs loadings: flexure modulus of elasticity (*E*), flexure strength (σ_u_), and flexure toughness at 5% strain level (*T*-5%).

COOH-MWCNTs	Criterion	*E* (GPa)	σ_u_ (MPa)	*T*-5% (MPa.mm/mm)
0 wt% (neat)	Mean	6.73	147.7	5.85
	STD	0.29	0.58	0.03
0.5 wt%	Mean (% increase)	7.00 (4%)	167.7 (13%)	6.05 (3.53%)
	STD	0.26	4.04	0.21
1.0 wt%	Mean (% increase)	7.60 (13%)	179 (21%)	6.74 (15.28%)
	STD	0.78	6.08	0.4
1.5 wt%	Mean (% increase)	8.00 (19%)	189 (28%)	7.06 (20.70%)
	STD	0.46	9.54	0.42

Previous research reported that significant damage in the matrix occurs prior to fiber reorientation and breakage when carbon woven fabric composites are loaded off-axis [32,33]. In this case, the damage in the matrix is gradual, causing significant nonlinearity in the load-displacement response. Similarly, the failure behavior of the off-axis flexure test can be therefore attributed to the damage of the epoxy matrix; thus, the effect of the multi-wall carbon nanotubes is evident. Similar findings on the significance of carbon nanotubes on the on- and off-axis tension test of composite coupons with carbon nanotubes were reported elsewhere by the authors [32]. It can be argued that the improvement in the off-axis behavior is attributed to a strong bond between the COOH functional groups attached to the MWCNTs and the epoxy groups. This bond seems to significantly improve the matrix behavior and, thus, to improve the flexure strength and modulus of the composite.

**Figure 6:**
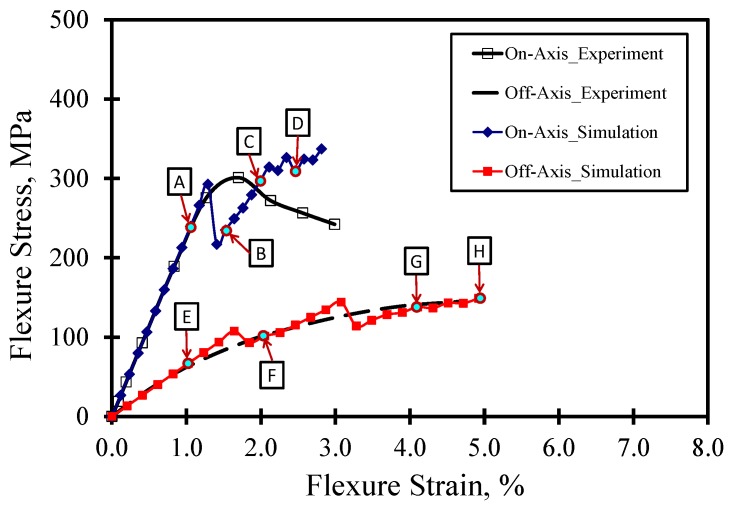
Comparison between experiment and FE simulation for on- and off-axis flexure behavior.

Figure 6 shows a comparison between the stress-strain curves for the on- and off-axis flexure test obtained experimentally and numerically. In general, the figure shows fair agreement between the experiments and FE simulations. However, the FE simulation fails to predict the on-axis post-peak behavior. This can be attributed to the fact that MCT is a class of FE simulation that uses a representative volume element (RVE) based on idealizing the microstructure of woven composites to develop a unit cell. The unit cell is used in the analysis and the average stress for each constituent is checked. Therefore, the FE model does not capture severe damage associated with large deformations and any change in geometry due to fiber breakage in compression. In order to capture these effects, the complete non-linear stress-strain curves for the constituents would help in improving the prediction of the post-peak behavior. This might be outside the scope of this effort, which is concerned with identifying the composite constituent responsible for failure. Furthermore, the numerical off-axis simulation exhibited multiple drops and subsequent increases of load in a “saw-tooth”-like behavior in the stress-strain, as reported elsewhere [29]. Such behavior depends on the FE mesh size and could be attributed to matrix damage in some elements and subsequent stress redistribution to undamaged elements.

**Figure 7:**
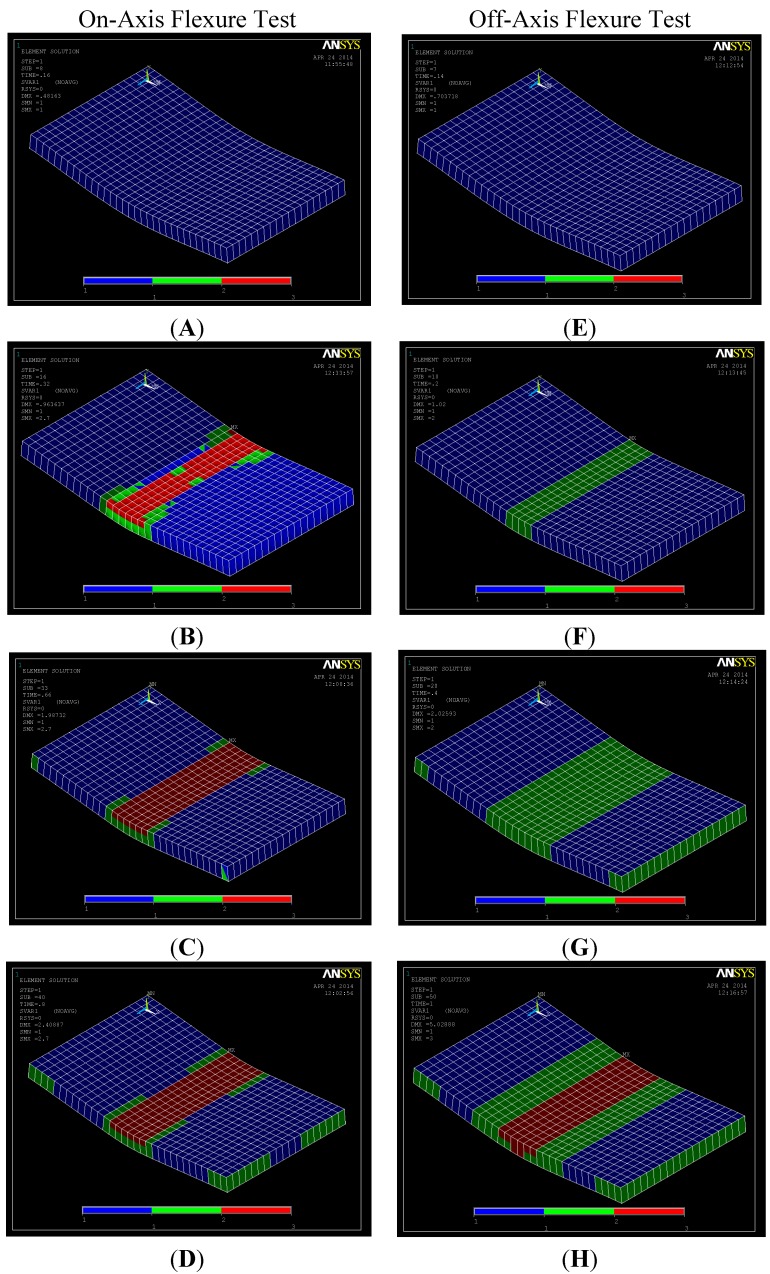
Contour plots for undamaged/damaged matrix and fibers for on- and off-axis flexure test at different strain level. (**A**) ~1.0%; (**B**) ~1.5%; (**C**) ~2.0%; (**D**) ~2.5%; (**E**) ~1.0%; (**F**) ~2.0%; (**G**) ~4.0%; (**H**) ~5.0%.

Notes:  Undamaged matrix and undamaged fiber;  Failed matrix and undamaged fiber;  Failed matrix and failed fiber.

On the other hand, the FE simulation is capable of predicting the off-axis flexure behavior to a great extent. Snapshots for the undamaged and damaged regions at different strain levels are shown in Figure 7. The images from A–E correspond to the on-axis test results, while the images from F–J correspond to the off-axis test results. For the on-axis test, it is observed that significant fiber damage occurs at the 1.5% strain level in the compression zone only. As the strain increases, the fiber and matrix damage propagate. On the other hand, significant matrix damage is observed in the off-axis test at the 3% strain level prior to any fiber damage. The onset of fiber damage is observed at a relatively high strain level (4.0%) in the compression zone. The fiber damage extends through the depth at the 5.0% strain level. The FE simulation confirms the fiber domination in the on-axis flexure behavior and the matrix domination in the off-axis flexure behavior, as shown in Figure 7.

**Figure 8:**
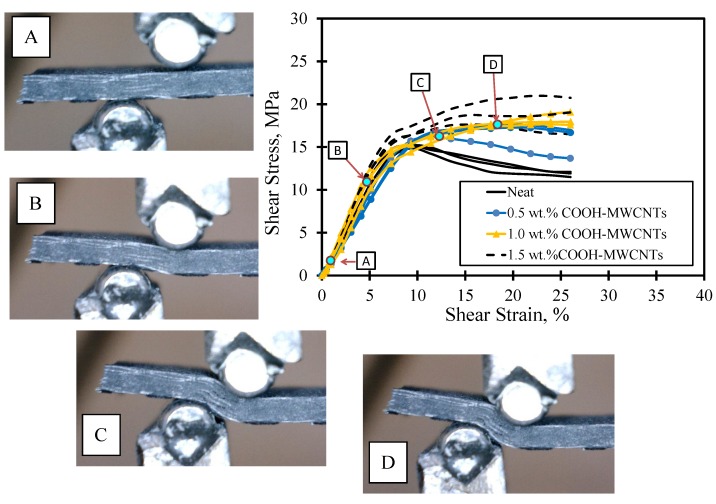
Shear stress-strain curves for composite plates with *in situ* microscopic images.

**Figure 9:**
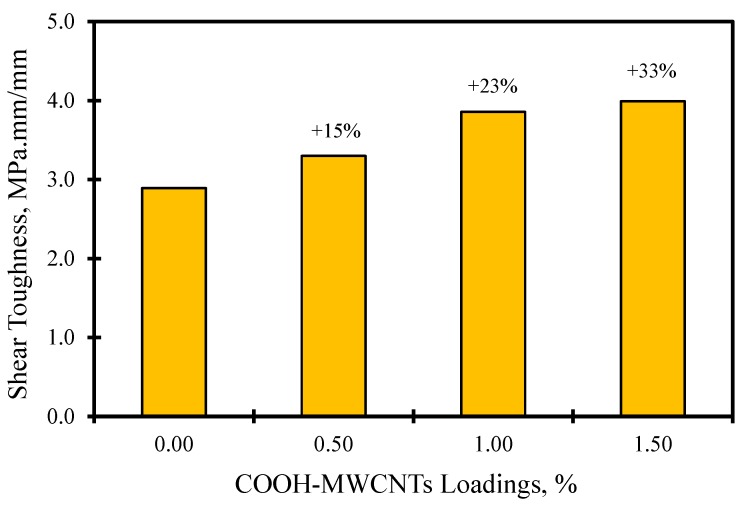
Comparison of shear toughness at the 25% strain level for various wt% of COOH-MWCNTs.

The shear stress-strain response of FRP composite plates with various contents of MWCNTs is also shown in Figure 8. The figure also displays *in situ* microscopic images of the deformation at the loaded part. The images show that no cracks were observed, as the stress was within the linear elastic zone. As the stress increased passing the linear zone, delamination between the CFRP laminates took place. In the stress-strain curve, it can be noted that the neat epoxy CFRP composites undergo a strain softening plateau after passing the linear elastic zone. This is contrary to the majority of the COOH-MWCNT-reinforced epoxy CFRP composites, where a clear trend of the straining hardening plateau occurred after passing the linear elastic zone. We attribute the difference in the shear behavior to the improvements in the interlaminar shear strength of the epoxy matrix in the composites. Such an improvement might be explained by the chemical reaction of the functionalized COOH-MWCNTs and the base resin. To further quantify the improvement associated with the addition of COOH-MWCNTs on the shear response, the shear toughness up to the 25% strain level is computed and compared in Figure 9. The average shear toughness was calculated for the neat, 0.5 wt%, 1.0 wt% and 1.5 wt% COOH-MWCNT cases as 2.89, 3.30, 3.86 and 3.39 MPa.mm/mm respectively. A noticeable 33% increase in the shear toughness is associated with the addition of 1.5 wt% COOH-MWCNTs compared to neat composite. This increase might be attributed to the significant increase in the inelastic energy absorption due to the strain hardening plateau associated with the addition of COOH-MWCNTs, as is apparent in Figure 8.

**Figure 10:**
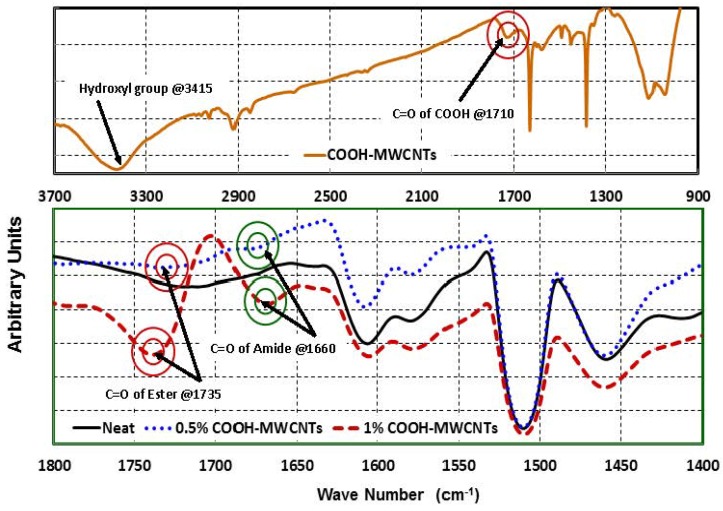
FTIR transmission curves of (**a**) COOH-MWCNTs powder and (**b**) COOH-MWCNTs/epoxy nanocomposites.

Analysis of the FTIR measurements shown in Figure 10 was performed. In Figure 10a, the characteristic vibrational modes of the carbonyl group (1710 cm^−1^) and the hydroxyl group (3415 cm^−1^) were observed in the spectrum of the powder-functionalized COOH-MWCNTs. We look at three cases in Figure 10b: neat epoxy, epoxy with 0.5 wt% COOH-MWCNTs and epoxy with 1 wt% COOH-MWCNTs. For the COOH-MWCNTs/epoxy, C=O stretching vibration peak (red) of the ester groups is observed at 1735 cm^−1^, while the C=O stretching vibration peak (green) of the amide groups is at 1660 cm^−1^, formed by the reaction of the carboxylic group in COOH-MWCNTs with the amine-based hardener. The amide groups peak at 1660 cm^−1^ and the ester group peak at 1735 cm^−1^ are weakly apparent in 0.5 wt% COOH-MWCNTs and strongly apparent in 1 wt% COOH-MWCNTs, and are all missing in the neat epoxy.

The above peaks can be explained by the fact that in neat epoxy, the carbonyl group peak is all missing, due to the absence of the COOH group and the complete reaction of epoxy. In the presence of COOH-MWCNTs, part of the carboxyl groups reacted with the epoxy base in the first step of fabrication and produced ester showing the C=O red peak, which is shifted to be at 1735 cm^−1^. This shift in the C=O peak due to COOH has been reported in the literature by Zou *et al.* [34] and Kim *et al.* [35]. This reaction might have happened via direct coupling between carboxylic group COOH of the COOH-MWCNTs and the hydroxyl groups of the epoxy resin. The potential for such a direct reaction has been reported by Kim *et al.* [36] and can be attributed to the fact that ester formation is feasible when the stoichiometric ratio is shifted to let one component of the reactants to present in a large excess. Since the epoxy resin is in a very large excess compared with COOH, this enhances the chances of the reversible esterification reaction in the forward direction according to Le Chatelier’s principle. It is also believed that the conditions of mixing COOH-MWCNTs with the epoxy resin under relatively high temperature, 40 °C for one hour then 80 °C for 2 h, would enable the removal of the small amount of water formed during esterification, which would further favor the forward esterification reaction.

In the second step of nanocomposite fabrication, the amine-based hardener was added to the epoxy base/COOH-MWCNTs dispersion. Therefore, all of the remaining carboxyl groups reacted with the hardener during the curing reaction of the epoxy and formed amide groups, which showed their C=O blue peak at 1660 cm^−1^, as reported previously by Zhang *et al.* [37]. The FTIR observations prove our argument that the COOH functional group resulted in the reaction between the MWCNTs and the epoxy and, thus, improved the shear strength of the epoxy matrix and the bond strength of the COOH-MWCNTs/epoxy nanocomposite matrix and carbon fibers. These improvements, in turn, resulted in the observed strength, modulus and ductility improvements of the flexural and shear off-axis behavior of CFRP.

Finally, it is evident from this study that improvements of 20%–30% can be achieved in the flexure properties of woven fabric composites by incorporating 1.5 wt% COOH-MWCNTs in epoxy. Moreover, improvements reaching 45% in the post peak energy of composites can be achieved with the addition of COOH-MWCNTs. These improvements in strength and toughness can be of significant value for woven fabric composite applications governed by the shear strength of the polymer matrix. Examples include composite pipelines, armored vehicles, aircrafts/aerospace shuttles and offshore structures. Delamination and debonding in these structures due to blasts, environmental condition or cyclic loading are critical parameters in design.

## 4. Conclusions

In this experimental investigation, the flexure and shear behavior of the multi-scale on-axis and off-axis functionalized COOH-MWCNTs/epoxy woven carbon fabric composite plates were examined. The results showed that the flexure behavior of such thin woven fabric composite plates depends significantly on the fiber orientation. Statistically-significant improvements in the mechanical properties were observed in the off-axis flexure behavior of CFRP incorporating COOH-MWCNTs, while no significant statistical difference was observed when the flexure composite plates are loaded on-axis. By using the 1.5 wt% COOH-MWCNTs/epoxy nanocomposite, flexure strength, modulus and toughness improvements of 28%, 19% and 20.7% were achieved. FE simulation showed that the fiber dominates the on-axis flexure behavior, while the matrix dominates the off-axis flexure behavior. Furthermore, this investigation also showed that more ductile woven carbon composite plates can be obtained by incorporating COOH-MWCNTs. The improvement in the toughness can reach 33% with the addition of 1.5 wt% COOH-MWCNTs. Microstructural investigations using FTIR showed that the mechanical improvements observed might be attributed to the chemical reaction of the MWCNTs with epoxy through the COOH functional group. Such a reaction resulted in the improved shear strength of the polymer matrix and the improved bond strength between the matrix and the carbon fibers. The above improvement in mechanical properties using COOH-MWNCTs can benefit several composite applications.
